# Focal Cerebral Ischemia Induces Expression of Glutaminyl Cyclase along with Downstream Molecular and Cellular Inflammatory Responses

**DOI:** 10.3390/cells13171412

**Published:** 2024-08-23

**Authors:** Corinna Höfling, Luise Ulrich, Sina Burghardt, Philippa Donkersloot, Michael Opitz, Stefanie Geissler, Stephan Schilling, Holger Cynis, Dominik Michalski, Steffen Roßner

**Affiliations:** 1 Paul Flechsig Institute for Brain Research, University of Leipzig, 04103 Leipzig, Germany; corinna.hoefling@medizin.uni-leipzig.de (C.H.);; 2 Department of Neurology, University of Leipzig, 04103 Leipzig, Germany; dominik.michalski@medizin.uni-leipzig.de; 3 Fraunhofer Institute for Cell Therapy and Immunology, Department of Molecular Drug Design and Target Validation, 06120 Halle, Germany; stefanie.geissler@izi.fraunhofer.de (S.G.); stephan.schilling@izi.fraunhofer.de (S.S.); holger.cynis@izi.fraunhofer.de (H.C.); 4 Faculty of Applied Biosciences and Process Engineering, Anhalt University of Applied Sciences, 06366 Köthen, Germany; 5 Junior Research Group, Immunomodulation in Pathophysiological Processes, Faculty of Medicine, Martin-Luther-University Halle-Wittenberg, Weinbergweg 22, 06120 Halle (Saale), Germany

**Keywords:** CCL2, experimental stroke, glutaminyl cyclase, microglia, astrocytes

## Abstract

Glutaminyl cyclase (QC) and its isoenzyme (isoQC) catalyze the formation of N-terminal pyroglutamate (pGlu) from glutamine on a number of neuropeptides, peptide hormones and chemokines. Chemokines of the C-C ligand (CCL) motif family are known to contribute to inflammation in neurodegenerative conditions. Here, we used a model of transient focal cerebral ischemia to explore functional, cellular and molecular responses to ischemia in mice lacking genes for QC, isoQC and their substrate CCL2. Mice of the different genotypes were evaluated for functional consequences of stroke, infarct volume, activation of glia cells, and for QC, isoQC and CCL2 expression. The number of QC-immunoreactive, but not of isoQC-immunoreactive, neurons increased robustly in the infarct area at 24 and 72 h after ischemia. In parallel, immunohistochemical signals for the QC substrate CCL2 increased from 24 to 72 h after ischemia induction without differences between genotypes analyzed. The increase in CCL2 was accompanied by morphological activation of Iba1-immunoreactive microglia and recruitment of MHC-II-positive cells at 72 h after ischemia. Among other chemokines quantified in the brain tissue, CCL17 showed higher concentrations at 72 h compared to 24 h after ischemia. Collectively, these data suggest a critical role for QC in inflammatory processes in the stroke-affected brain.

## 1. Introduction

The enzyme glutaminyl cyclase (QC; E.C. 2.3.2.5) catalyzes the conversion of N-terminal glutamine into pyroglutamate (pGlu) [1,2]. In mammals, QC was found to be abundant in the hypothalamus, pituitary and adrenal medulla, but also in lymphocytes [1,3,4]. Physiological QC substrates are proteins that reside in these brain structures and include, amongst others, thyrotropin releasing hormone (TRH), orexin A and neurotensin [5]. In addition, in the human and mouse brain, QC is expressed in distinct regions such as the Edinger–Westphal nucleus, locus coeruleus, nucleus basalis [6], hippocampus [7] and substantia nigra [8]. Intriguingly, these brain regions are affected in Alzheimer’s disease (AD) and in Parkinson’s disease, and QC was demonstrated to be implicated in the pGlu modification of pathogenic amyloid-β (Aβ) and α-synuclein (aSyn) proteins [6,7,8,9]. QC was also found to be a regulator of huntingtin neurotoxicity by a mechanism that involves αB-crystallin [10]. Pharmacologic inhibition and genetic ablation of QC activity in AD transgenic animal models reduced pGlu-Aβ generation and total Aβ load and ameliorated learning and memory deficits [9,11,12,13]. In addition, completed and ongoing AD clinical trials indicated safety, tolerability and efficacy of the QC inhibitor PQ912 in human subjects [14,15,16]. 

The expression of the isoenzyme of QC (isoQC; E.C. 2.3.2.5) in the brain is more widespread than that of QC [5,17,18]. IsoQC localization to the Golgi apparatus [19] determines its substrate specificity. For example, hormones such as TRH, which mature in secretory granules by the enzymatic activity of prohormone convertases, cannot be pGlu-modified by Golgi-resident isoQC. In contrast, proteins with N-terminal glutamine already generated in the endoplasmic reticulum, such as gonadotropin-releasing hormone and the chemokine CCL2, represent isoQC substrates [5]. Indeed, isoQC was demonstrated to account for pGlu modification of CCL2 and subsequent monocyte infiltration in the model of thioglycolate-induced peritonitis and for atherosclerotic pathology in ApoE3*Leiden mice [20]. In contrast, QC, but not isoQC, was found to be co-regulated with CCL2 and CX3CL1 in human endothelial and smooth muscle cells [21]. Moreover, application of dual QC/isoQC inhibitors alleviated inflammation in mouse models of non-alcoholic fatty liver disease [22] and septic arthritis [23] by destabilizing CCL2. 

Thus, the QC/isoQC-CCL2 axis seems to have a pivotal role in several inflammatory processes. Based on observations in traumatic brain injury [24], epilepsy [25], tumors [26,27], AD [18,28,29] and stroke [30,31], CCL2 is also implicated in neuroinflammation. However, a potential regulation of CCL2 stabilization and bioactivity under neuroinflammatory conditions by QC/isoQC was not yet investigated. Therefore, we here used a model of transient focal cerebral ischemia, which is known to be associated with inflammatory processes, in genetically altered mice to explore the impact of QC, isoQC and CCL2 expression in experimental stroke, as well as the consequences of the genetic ablation on the functional outcome, infarct volumes, the reactions of diverse cell types and biochemical consequences.

## 2. Materials and Methods

### 2.1. Characterization of KO Mice

QC KO mice were generated by homologue recombination leading to deletion of exons 4 and 5 of the QC gene [32]. To establish isoQC KO mice, a targeted point mutation (T>A; L144X) was introduced into the isoQC gene, which leads to a stop of mRNA translation [5]. The CCL2 KO mouse line was generated by deletion in exon 2 and insertion of a stop codon in exon 1 [33]. Mice of all KO genotypes were found to be viable and fertile with the same body weight as wild-type mice and did not show deficits in motor function, cognitive performance and activity. The only KO effect observed was a slight hypothyroidism in the absence of hypogonadism in the QC KO strain [32]. CCL2 KO mice were purchased from Jackson Lab, Bar Harbor, ME, USA. QC and isoQC KO mice were generated by and bred with permission of Vivoryon Therapeutics (Probiodrug AG), Halle, Germany. All mouse lines were maintained on a C57Bl/6J background at the Fraunhofer Institute for Cell Therapy and Immunology, Halle, Germany, at 12 h day/12 h night cycles with food and water ad libitum in individually ventilated cages that contained nest building material.

### 2.2. Genotyping

Genotyping was performed according to a standard DNA isolation and PCR protocol essentially as described elsewhere [34]. Briefly, DNA was isolated from ear punches using the 1-Step kit (Nexttec, Leverkusen, Germany). DNA was analyzed for the presence of respective KOs by PCR using specific primers shown in Table 1. Whereas mixes of 3 different primers directly reveal QC KO and CCL2 KO, the PCR product of isoQC-specific primers was further digested using AvrII leading to 2 distinct bands in case of the presence of at least one isoQC KO allele. Samples were analyzed on a 1.6% agarose gel in TAE buffer.

### 2.3. Experimental Stroke

The experiments have been performed following the 3R principles and ARRIVE guidelines. Induction of transient focal cerebral ischemia was approved by the Ethical Committee for Animal Research of Landesdirektion Sachsen, Germany, license number TVV 13/18. Seven days before experimental stroke, mice were transferred to the Animal Care Facility of the Medical Faculty, Leipzig University, and trained to be fed with a “high calorie dietary supplement for laboratory rodents” (DietGel® Boost, Clear H_2_O, Portland, ME, USA) from a syringe. Transient focal cerebral ischemia in 4 months old male mice of different genotypes with an initial body weight of 28.3 g was performed by the filament-based model as originally described in [35], with some minor adaptions. Briefly, under anesthesia (Fentanyl, 0.05 mg/kg; Midazolam, 5 mg/kg; Medetomidin 0.5 mg/kg) a standardized silicon-coated monofilament of 0.23 mm diameter (Doccol, Sharon, MA, USA) was introduced into the right carotid artery and moved forward to the origin of the middle cerebral artery, and kept in place for 30 min. This procedure resulted in focal ischemia typically involving subcortical regions of the middle cerebral artery territory and, at least in part, an involvement of neocortical areas. Pain management included local anesthesia at the medial site of the neck and the use of lidocaine (Licain, DeltaSelect, Dreieich, Germany). We decided to use male mice only in our study. We are aware of this weakness in the experimental design but including females would not only double the animal number but also introduce the estrous cycle as an additional variable. Therefore, and considering 3R guidelines, females were not included in this study.

After observation periods of 24 or 72 h, mice were sacrificed by CO_2_ inhalation and the brain tissue was prepared for immunohistochemical or biochemical analyses. Overall, 58 wild-type, 41 QC KO, 48 isoQC KO, and 50 CCL2 KO mice were subjected to ischemia, finally leading to a group size of *n* = 6 each for biochemical and for histological analyses at 24 and 72 h after induction of ischemia. The post-ischemia intervals of 24 and 72 h were chosen to monitor neuronal cell death, the expression of chemokines and the activation/recruitment of microglial cells and astrocytes in the acute ischemia phase. Additionally, 12 mice of each genotype without ischemia served as controls for biochemical (*n* = 6) and histological analyses (*n* = 6). The group size was calculated based on the estimated effect size of QC/isoQC KO on CCL2 concentrations and glia activation of 1.67 [36] and an error of 0.05 to reach a power of 0.8.

### 2.4. Evaluation of Functional Outcome

The investigators were blind to the group allocation during the experiment and when scoring the functional outcome. The Menzies score [37] is a clinical score to evaluate neurobehavioral deficits without relevant manipulation. Thereby, the degree of motoric deficits is recorded as follows: 0 = “no apparent deficits”, 1 = “contralateral forelimb flexion”, 2 = “decreased grip of the contralateral forelimb while tail pulled”, 3 = “spontaneous movement in all directions, contralateral circling only if pulled by tail”, 4 = “spontaneous contralateral circling”. Animals were scored every 24 h after re-perfusion. At the same time points, animal weight was taken. In case of loss of more than 20% of initial body weight, the animals had to be excluded from further study procedures and were sacrificed. As an additional outcome, the “overall physical condition” was calculated. This is based on body weight, behavior and mobility, appearance and Menzies score, and thus, gives an estimate of the animals’ wellbeing. 

#### Overall Physical Condition

As an additional outcome parameter, the overall physical condition was recorded while considering body weight, behavior and mobility, general appearance, and Menzies score. Each parameter was scored from 0 to 3, with 3 being the worst. Score 0 included active behavior, smooth, shiny coat, a weight loss of less than 5%, and a Menzies score of 0–2. Score 1 resulted from decreased reaction, dull coat, and soiled eye corners, a weight loss of less than 13%, and a Menzies score of 3. Score 3 included chewing posture and arched back, stuck body openings, and shaggy coat, a weight loss of less than 20%, and a Menzies score of 4. Score 3 in any of the parameters examined was also the termination criterion and included no response, apathy, and no locomotion in behavior and mobility. In appearance, score 3 was achieved when the animal had closed eyes and was cool. 

### 2.5. Mouse Plasma and Brain Tissue Preparation

After the sacrifice, a small volume (about 0.5 mL) of blood was collected by cardiac puncture, which was transferred to heparin-coated vials and centrifuged immediately at 7000× *g* for 10 min at 4 °C. Plasma was carefully removed from the cell pellet and stored at −80 °C.

For histochemical analyses, mice were transcardially perfused with 0.9% saline followed by perfusion with 4% paraformaldehyde in phosphate buffer (0.1 M, pH 7.4). The brains were removed from the skull and post-fixed by immersion in the same fixative overnight at 4 °C. After cryoprotection in 30% sucrose in 0.1 M phosphate buffer for three days, 30 µm thick coronal sections were cut on a sliding microtome and collected in phosphate buffer supplemented with 0.025% sodium azide for storage. 

For biochemical analyses, mice were transcardially perfused with 0.9% saline, the brains were removed from the skull, the cerebellum was cut off and the cerebrum was dissected into the right (ischemic) and left (control) hemispheres, frozen on dry ice and stored at −80 °C.

### 2.6. Calculation of Infarct Volumes

The infarct volume was determined by using every 9th coronal brain section for immunohistochemical NeuN + HuC/D labeling of neurons and subsequent calculation of the infarct area, which was integrated over the rostro-caudal infarct area extension. Details on the respective image analyses are given below. 

### 2.7. Immunohistochemistry

In the present study, the following primary antibodies against neuronal, glial and inflammatory marker proteins were used (Table 2). The specificity of the antisera against QC, isoQC and CCL2 was verified in the brain tissue of the respective KO mice by the absence of the typical immunoreactivity present in the wild-type mouse brain (Supplementary Figure S1).

#### 2.7.1. Single Labeling of NeuN and HuC/D, QC, isoQC and CCL2 in Mouse Brain Sections

In order to detect significant neuronal populations in the brains of wild-type, QC KO, isoQC KO and CCL2 KO mice, single labeling immunohistochemistry with mouse antibodies against NeuN and HuC/D was performed on free-floating coronal brain sections. In addition, the expression of CCL2 and its modifying enzymes, QC and isoQC, was revealed by immunohistochemical single labeling using primary goat or rabbit antibodies (Table 2). Brain sections were washed in 0.1 M phosphate buffer (pH 7.4) for 5 min and endogenous peroxidases were inactivated by treating brain slices with 60% methanol containing 1% H_2_O_2_ for 60 min followed by three washing steps with Tris-buffered saline (TBS, 0.1 M, pH 7.4) for 5 min each. After masking unspecific binding sites with blocking solution (3% donkey anti-mouse IgG Fab fragment, 2% normal donkey serum in TBS containing 0.3% Triton X-100) for 60 min, sections were incubated in 5% normal donkey serum in TBS containing 0.1% Triton-X-100 with the primary antibodies as indicated in Table 2 for 24 h at 4 °C. Brain sections were then washed three times in TBS for 5 min each before being incubated with biotinylated secondary donkey anti-mouse or donkey anti-goat or donkey anti-rabbit antibodies (Dianova, Hamburg, Germany; 1:1000) in TBS containing 2% bovine serum albumin (BSA) for 60 min. After three washing steps in TBS for 5 min each, slices were incubated with ExtrAvidin peroxidase (Sigma, Neustadt, Germany; 1:2000) in TBS/2% BSA followed by washing steps and pre-incubation in Tris buffer (0.05 M, pH 7.6) for 5 min. Finally, visualization of peroxidase binding was performed by incubation with 4 mg 3,3’-diaminobenzidine (DAB) and 2.5 µL 30% H_2_O_2_ per 5 mL Tris buffer. After washing, sections were mounted onto glass slides and cover slipped.

#### 2.7.2. Double and Triple Immunofluorescent Labeling in the Mouse Brain

In order to reveal the activation of microglia cells and astrocytes in the infarct area and the cell type-specific expression of CCL2 in ischemic mouse brain tissue, cocktails of primary antibodies from different species were used as specified in Table 2. Brain sections were incubated with cocktails of primary antibodies for 24 h at 4 °C. Sections were then washed three times with TBS followed by incubation with cocktails of Cy2-, Cy3- or Cy5-conjugated donkey anti-mouse, -rabbit, -guinea pig or -goat antisera, respectively, (1:200 each; Dianova, Hamburg, Germany) in TBS containing 2% BSA for 60 min at room temperature. After washing, sections were mounted onto glass slides and cover slipped. Switching the fluorescent labels of the secondary antibodies generated similar results as when following the procedure outlined above. For all single, double and triple immunohistochemical labelinglabeling in the brain sections described above, control experiments in the absence of primary antibodies were carried out. In each case, this resulted in unstained brain sections. In addition, the specificity of the primary antibodies against QC, isoQC and CCL2 was validated in non-ischemic and ischemic brain tissue of wild-type and the respective KO genotype (Supplementary Figure S1).

### 2.8. Microscopy

#### 2.8.1. Light Microscopy

Mouse brain tissue sections immunohistochemically stained with DAB or DAB-Ni for NeuN + HuC/D, QC, isoQC and for MHC-II expression were examined with an Axio-Scan.Z1 slide scanner connected with a Colibri.7 light source and a Hitachi HV-F202SCL camera (Zeiss, Göttingen, Germany). High resolution images of brain sections were taken using a 20× objective lens with 0.8 numerical aperture (Zeiss). Images were digitized by means of ZEN blue 2.6 software and analyzed using the ZEN imaging tool. 

#### 2.8.2. Fluorescence Microscopy

Brain tissue sections of wild-type and KO mice immunochemically stained with Cyanine labeled secondary antibodies were examined with an AxioScan.Z1 microscope (Zeiss, Göttingen, Germany) equipped with a Colibri 7 light source including 6 LED to cover the light spectrum from 385 to 735 nm to generate seven fluorescence excitation lines for the samples to be analyzed. All LEDs besides the green/yellow line (combined 555/590 nm filter changer) come with individual excitation filters. Emission filters select for Cy2 at 524 nm, for Cy3 at 595 nm and Cy5 at 676 nm and Hoechst 33,342 at 425 nm. Pictures were taken by means of Axiocam 506 and a Plan-Apochromat objective (20×/0.8) and exposure time of 15 ms for Cy2, 20 ms for Cy3, 30 ms for Cy5 and 2 ms for Hoechst 33,342. Images were digitized by means of ZEN blue 2.6 software.

#### 2.8.3. Image Analysis

Images were analyzed by means of trainable segmentation with Apeer Machine Learning toolkit and ZEN blue 3.5 Intellesis software (Zeiss, Göttingen, Germany). By using machine learning, errors and influences of user bias were avoided. For single neuronal staining, the area missing NeuN and HuC/D immunoreactivity was determined manually and calculated by ZEN software. For QC-, isoQC- and MHC-II-positive cells the number of these cells (QC, MHC-II) or the staining intensity (isoQC) was determined in defined regions of interest. For quantification of fluorescence signals, trainable segmentation was applied to recognize specific fluorescence signals from CCL2, NFL, GFAP, CD68, TMEM119 and Iba1 separately. The final readout for NFL, GFAP, CD68 and TMEM119 was the area covered by positive signals. For CCL2, the number of positive signals was determined independent of signal size. For Iba1 staining, the training was aimed to recognize the number of morphological reactive microglia (enlarged cell body, less branches), but not surveying microglia (small cell body, long branches). During analysis, the number of these cells per brain slice was determined. The choice of the quantification method was based on the applicability of the respective procedure. For example, isoQC is a Golgi-resident, non-secreted enzyme where the immunohistochemical signal arises from neuronal labeling and signal intensity is related to neuronal numbers. In contrast, QC is a secreted enzyme and measuring signal intensity would not give an accurate quantification of QC-positive neurons. Therefore, QC-positive neurons were counted.

### 2.9. Quantification of Chemokines in Brain Tissue and Plasma 

#### 2.9.1. Cerebrum Preparation

In order to prepare the mouse cerebrum for Bio-Plex analysis, the left and right cerebrum of the ischemic group and right cerebrum of control mice were homogenized in T-Per buffer (Tissue Protein Extraction Reagent, Thermo Fisher Scientific, Darmstadt, Germany) at a concentration of 333 mg/mL with Protease Inhibitor Cocktail Tablets (Merck, Darmstadt, Germany) by using a Precellys homogenizer (VWR, Dresden, Germany). The homogenate was centrifuged for 15 min at 10,000× *g* at 4 °C, kept on ice for transferring to 1.5 mL reaction tubes and centrifuged again for 30 min at 25,000× *g* at 4 °C. The soluble supernatant was collected, aliquoted and stored at −80 °C until use.

#### 2.9.2. BCA Protein Assay

The BCA assay (Pierce, Thermo Fisher Scientific; Darmstadt, Germany) was performed according to the manufacturer’s protocol. Cerebrum samples were 1:50 diluted in T-PER buffer. The albumin (BSA) standard working range corresponded to 20–2000 µg/mL (*n* = 2). Absorption was measured at 562 nm (Tecan Sunrise, Männedorf, Switzerland). 

#### 2.9.3. Bio Plex Analysis

For multiplex analysis of the plasma and cerebrum, an individually prepared 8-Plex (Bio-Rad, Feldkirchen, Germany) was used. The following cytokines were included: fractalkine/CX3CL1, IL-1β, IL-6, MCP-1/CCL2, MCP-5/CCL12, RANTES/CCL5, TARC/CCL17 and TNF-α. At the beginning, all desired cytokine beads were combined and 50 µL of the suspension was added per well to a 96-well plate. After washing twice, the samples (1:4 plasma diluted in Sample Diluent; 1:10 cerebrum diluted Sample Diluent containing 0.5% BSA), standards and blanks were transferred to the appropriate wells. Further steps of the implementation were made following the manufacturer’s instructions.

### 2.10. Statistical Analysis

To compare the ischemic with the control hemisphere, we used the Wilcoxon Signed Rank test. To compare differences between wild-type and the respective KO mice, the Mann–Whitney Rank Sum test was used. Statistical comparisons between all groups were evaluated using one-way ANOVA, with the Bonferroni post-hoc analysis. For calculations, Sigma Plot 14.0 (Systat Software Inc., Frankfurt/M., Germany) was used. Differences between groups were considered statistically significant for *p* values < 0.05.

## 3. Results

### 3.1. Consequences of Focal Cerebral Ischemia in Mice with Lacking Genes for QC, isoQC, and CCL2

**Figure 1:**
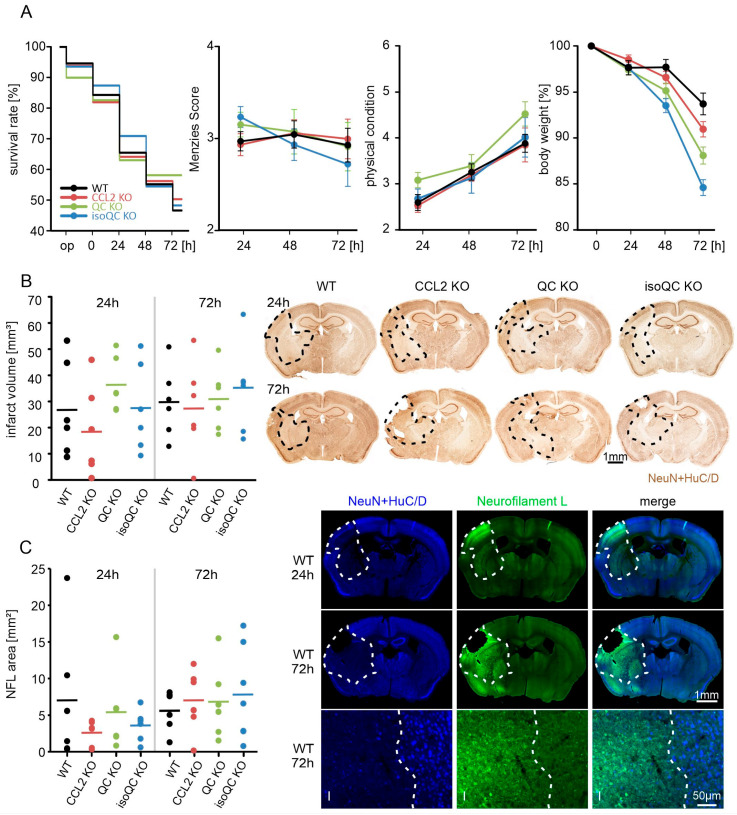
Characterization of focal cerebral ischemia in knock-out mouse models. Mice of different genotypes (wild-type, CCL2 KO, QC KO and isoQC KO) were subjected to transient focal cerebral ischemia for 30 min, followed by 24 h and 72 h observation periods. (**A**) Survival rate (*p* = 0.858), Menzies score (*p* = 0.91), overall physical condition (*p* = 0.82) and body weight (*p* = 0.78) did not differ significantly between genotypes. Only 45% (wild type) to 58% (QC KO) of the animals subjected to the operation procedure survived for 72 h. For all genotypes, a constant Menzies score of 3 was determined at all time points. In contrast, the overall physical condition steadily declined (higher scores indicating worse condition) during the 72 h post-surgery period, which was also characterized by a weight loss ranging between 7% in wild-type mice and 16% in isoQC KO mice. (**B**) Immunohistochemical NeuN + HuC/D images from experimental animals of all genotypes at 24 h and 72 h after onset of ischemia. The dashed lines indicate the infarct area identified by diminished NeuN + HuC/D labeling. The infarct volume was calculated from serial brain slices for all animals of all genotypes and was found to be between 18 mm^3^ (CCL2 KO, 24 h) and 35 mm^3^ (QC KO, 24 h). (**C**) As a complementary measure of infarct size, increased immunosignals for Neurofilament L (NFL) in the ischemic area was quantified in serial sections. Here, the smallest ischemic areas were detected for CCL2 KO mice at 24 h and largest for QC KO at 72 h after ischemia. Note the complementary loss of NeuN + HuC/D and induction of NFL immunoreactivity in the ischemic area (I).

Since the functional and cellular consequences of ischemia were not yet investigated in the QC KO and isoQC KO mouse brain, we first determined the survival rate, Menzies score, overall physical condition, body weight and infarct volume in all KO genotypes and wild-type mice (Figure 1). There were no statistically significant differences between the genotypes, but QC KO mice tended to provide the best survival rate and isoQC KO mice the largest body weight loss after ischemia (Figure 1A). The infarct volume was calculated from diminished NeuN + HuC/D labeling in brain sections demonstrating neuronal loss along the rostro-caudal extension of the ischemic area. There was a tendency of larger infarct volume by 72 h after ischemia onset compared to the 24 h time point, but there was also large inter-individual infarct size variation (Figure 1B). These data were corroborated by NFL labeling, which displayed increased immunoreactivity in brain regions affected by ischemia (Figure 1C).

**Figure 2:**
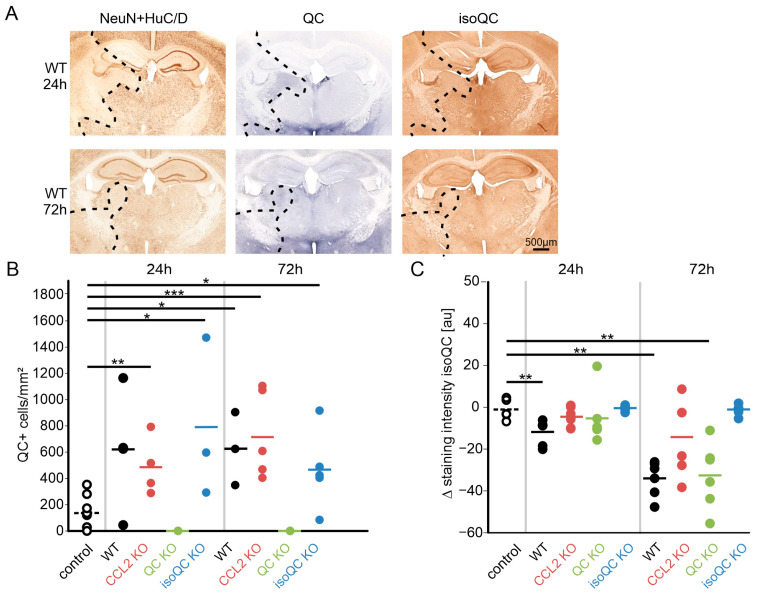
Expression of QC and isoQC in ischemic brain areas. (**A**) Immunohistochemical labeling for QC and isoQC in the wild-type mouse brain at 24 h and 72 h after ischemia. (**B**) There were significant increases in the numbers of QC-immunoreactive neurons in infarct areas identified by NeuN + HuC/D labeling in consecutive brain sections for all genotypes (except QC KO) at both survival time points. (**C**) In contrast, there was significantly reduced isoQC immunoreactivity in infarct areas compared to the non-ischemic hemisphere. * *p* < 0.05; ** *p* < 0.01; *** *p* < 0.001; differences statistically significant versus control.

Next, we analyzed the expression of the enzymes QC and isoQC in ischemic brain tissue. In order to relate the expression of both proteins to ischemia, they were detected in consecutive brain sections to those labelled for NeuN + HuC/D (Figure 2A). Interestingly, the expression of QC was increased in ischemic brain regions identified by absence of NeuN + HuC/D labeling, whereas the expression of isoQC was reduced (Figure 2B,C). Increased QC immunoreactivity was restricted to neurons and not observed in glial cells. There were no differences in the abundance of QC-positive neurons between wild-type and isoQC KO mice. Likewise, isoQC-immunoreactivity was not affected by QC KO. Notably, there was no QC immunoreactivity in the control and ischemic QC KO mouse brain and no isoQC immunoreactivity in the control and ischemic isoQC KO mouse brain (Supplementary Figure S1).

**Figure 3:**
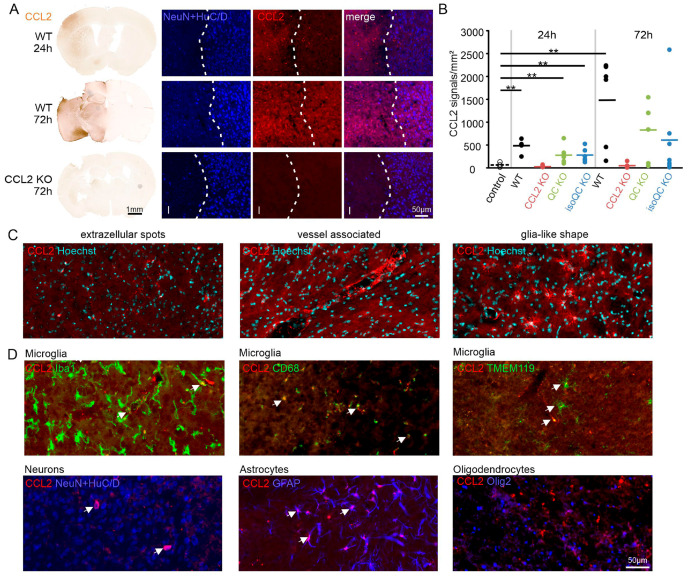
Quantification of CCL2 expression and co-localization to neuronal and glial markers. (**A**) Left: single immunohistochemical labeling of CCL2 in the wild-type (WT) mouse brain 24 h and 72 h after ischemia. Note the absence of CCL2 immunoreactivity in a CCL2 KO mouse brain 72 h after ischemia. Right: note the post-ischemia time-dependent increase in signal intensity and spreading of the CCL2 immunosignal beyond the infarct (I) region demarked by the dashed line related to reduced NeuN + HuC/D labeling at 72 h. (**B**) Quantification of CCL2 immunosignals demonstrating increased CCL2 in the wild-type (WT) mouse brain at 24 and 72 h. Note that the mean values for CCL2 signals in QC KO and in isoQC KO are only half of the of the WT mice at both time points. (**C**) Appearance of CCL2 immunoreactivity in the ischemic cortex as extracellular spots, in association with vessels and in a glia-like shape. (**D**) Examples of double labeling of CCL2 with microglia markers Iba1, CD68 and TMEM119, with neurons (NeuN + HuC/D), astrocytes (GFAP) and oligodendrocytes (Olig2) in the ischemic cortex. Note the presence of CCL2 immunosignals in subsets of these neuronal and glial populations (arrows) except in oligodendrocytes. ** *p* < 0.01; differences statistically significant versus control.

We then quantified CCL2 immunohistochemical signals in ischemic brain tissue. CCL2 expression was absent in brain sections of control animals that did not experience ischemia (Supplementary Figure S1). In contrast, there was significant CCL2 immunoreactivity at the ischemic hemisphere at 24 h, and even more pronounced at 72 h after ischemia as shown by single DAB labeling (Figure 3A). This labeling was restricted to ischemic tissue at 24 h of ischemia, but also extended into neighboring non-ischemic brain areas at 72 h of ischemia as shown in double labeling with NeuN + HuC/D (Figure 3A). This is consistent with a CCL2 gradient from ischemic to non-ischemic brain tissue to recruit microglial cells and with a pro-inflammatory role of CCL2 in the clinical situation of stroke that might contribute to secondary neuronal cell death. Quantification of the CCL2 immunohistochemical signals validated this post lesion time-dependent increase in CCL2 and the absence in CCL2 in ischemic CCL2 KO mouse brain tissue (Figure 3B). In ischemic brain tissue of QC KO mice and of isoQC KO mice, the mean values of the CCL2 immunosignals were about half of those in in wild-type mice at both time points, although this reduction was not statistically significant (Figure 3B). Consistent with CCL2 being a secreted protein, its immunohistochemical appearance was mostly diffuse, but also present as extracellular spots, in vessel-associated and in glia-like shapes (Figure 3C). Double labeling with cell type-specific markers revealed a partial co-localization with the microglial markers Iba1, CD68 and TMEM119, with the neuronal markers NeuN + HuC/D and with the astrocyte marker GFAP, but not with the oligodendrocyte marker Olig2 (Figure 3D).

### 3.2. Consequences of Increased QC and CCL2 Expression in Ischemic Brain Tissue

**Figure 4:**
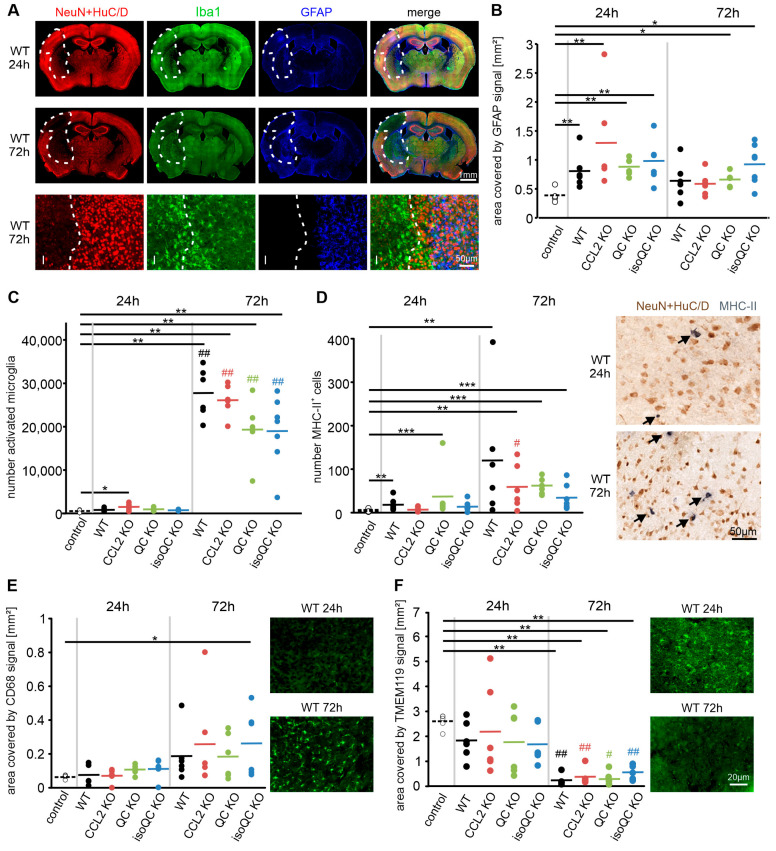
Glia cell activation/recruitment in ischemic brain regions. (**A**) Triple immunofluorescent labeling of the microglial marker Iba1 and the astrocyte marker GFAP in combination with NeuN + HuC/D to identify the cortical infarct area (I). In the high magnification images (bottom), the activation of Iba1-positive microglia in the ischemic region and the presence of GFAP-immunoreactive reactive astrocytes in the border zone is evident. (**B**) Quantification of GFAP immunosignals in brain sections of mice of different genotypes. Note the instant increase in GFAP expression at 24 h for all genotypes, which is not increase further at 72 h. (**C**,**D**) In contrast, both numbers of activated microglia (**C**) and of MHC-II cells (**D**) only increase at 72 h after ischemia for all genotypes. Note that the mean values for both cell types in QC KO mice and in isoQC KO mice are only half of that in wild-type (WT) mice. Arrows in (**D**) point towards MHC-II-positive neurons (black). (**E**,**F**) The immunoreactivity for CD68 is increased after ischemia in all genotypes (**E**), whereas TMEM119 immunosignals are reduced (**F**). * *p* < 0.05; ** *p* < 0.01; *** *p* < 0.001; differences statistically significant versus control. # *p* < 0.05; ## *p* < 0.01; differences statistically significant at 72 h versus 24 h.

In order to reveal the cellular responses to increased QC and CCL2 expression after ischemia, we performed triple labeling for NeuN + HuC/D, to identify the ischemic area, with the microglial marker Iba1 and the astrocyte marker GFAP (Figure 4A). While GFAP immunosignals were increased already by 24 h after ischemia and did not further increase by 72 h of ischemia (Figure 4B), the number of Iba1-positive morphologically activated microglia only increased by 72 h of ischemia (Figure 4C). Likewise, the number of infiltrating MHC-II-positive cells increased markedly by 72 h of ischemia (Figure 4D). Remarkably, and in analogy to the CCL2 immunosignals, the mean numbers of Iba1- and MHC-II-positive cells in the ischemic tissue of the QC KO and of isoQC KO mouse brain was only half of that in the wild-type mouse brain (Figure 4C,D). In addition, we observed a time-dependent increase in CD68 immunosignals (Figure 4E) and a converse decrease in TMEM119 immunosignals (Figure 4F). There were no statistically significant differences in the abundance of glial (sub)-types between wild-type mice and the KO mice under investigation in this study.

**Figure 5:**
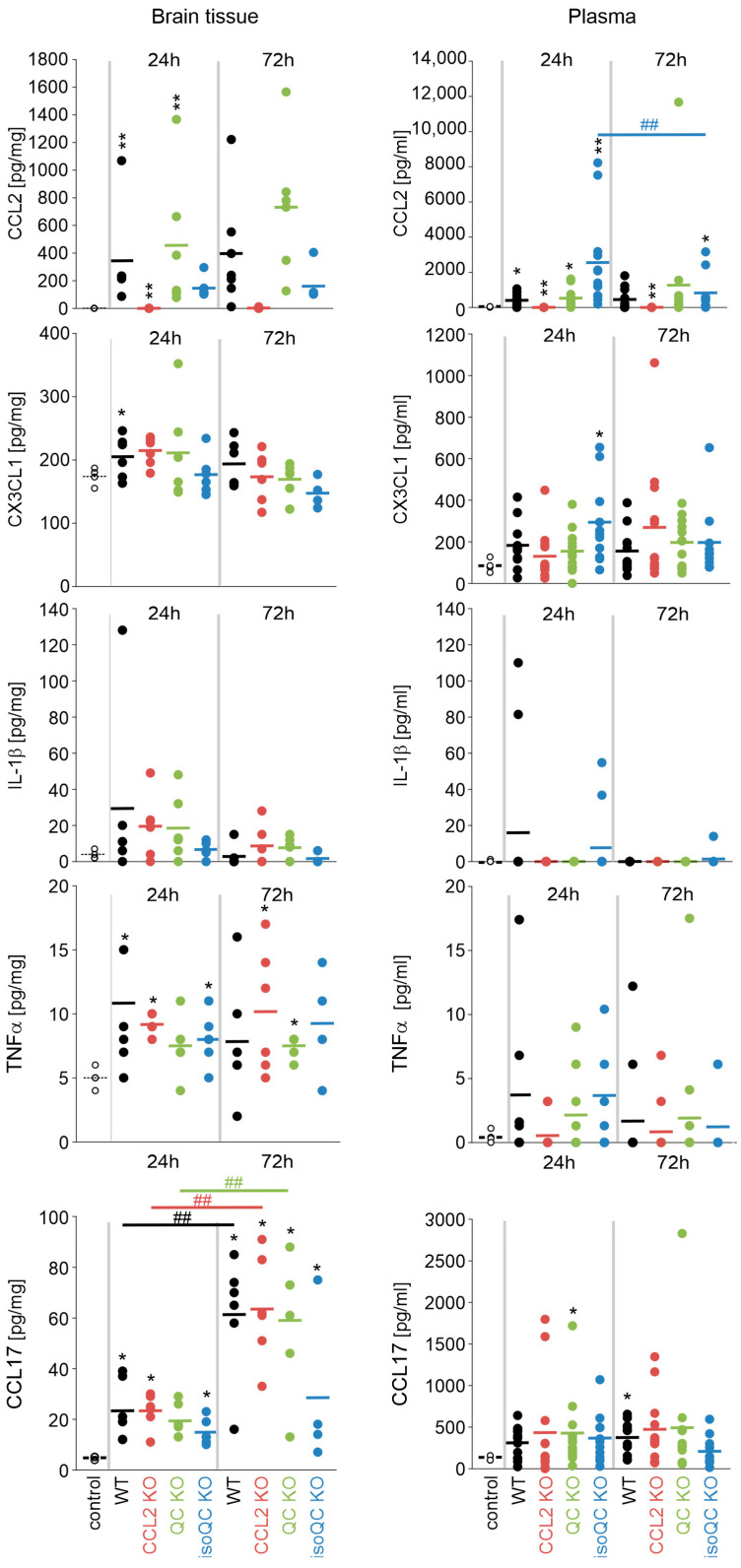
Quantification of chemokines in brain tissue and plasma of mice after ischemia. The concentrations of CCL2, CX3CL1, IL-1β, TNF-α and CCL17 were quantified by multiplex analyses in brain tissue of the ischemic hemisphere (**left**) and in plasma (**right**) at 24 h and 72 h after ischemia as indicated. Upregulation in the brain tissue is only selectively mirrored in the plasma, e.g., in case of CCL2. Please note the lack of CCL2 in CCL2 KO mice. * *p* < 0.05; ** *p* < 0.01; differences statistically significant versus control. ## *p* < 0.01; differences statistically significant at 72 h versus 24 h.

To obtain insights into inflammatory responses to ischemia, the concentrations of chemokines pre-selected from a broader panel based on their presence in ischemic brain tissue and in plasma were quantified by multiplex analyses in the brain tissue and plasma of experimental animals of all genotypes at both survival time points (Figure 5). At the primary site of pathology, the ischemic brain tissue, a time-dependent increase in CCL2 was detected, which was mirrored by increases of CCL2 in plasma in WT, QC KO and isoQC KO mice. In the brain tissue, there was a non-significant reduction in CCL2 in isoQC KO mice compared WT and QC KO mice, underlining the importance of isoQC for CCL2 maturation in the infarct area. Plasma levels of CCL2 did not further increase by 72 h post ischemia onset. Reduced CCL2 in isoQC mice was accompanied by trends of reduced levels of CX3CL1 and interleukin-1β in brain tissue 24 h post ischemia compared to the control tissue suggesting an impact on overall reduced inflammatory environment of isoQC KO at this time point. This effect was attenuated by 72 h post ischemia and was not consistently observed for the corresponding plasma chemokine concentrations. Interestingly, CCL17 levels increased between 24 h and 72 h of ischemia (Figure 5). In addition, at the early time point of 24 h, isoQC KO had a similar reducing effect on CCL17 as on CCL2 although CCL17 is not a known isoQC substrate.

## 4. Discussion

In the present study, we wanted to address the questions whether the expression of the CCL2-stabilizing enzymes QC and isoQC are influenced by ischemia in mouse brain and whether their absence in KO mice affects CCL2 concentrations and the downstream cellular and functional outcome after experimental stroke.

CCL2 is known to be induced in the brain by multiple events with an inflammatory component [24,26,28], including stroke [30,31]. In the CCL2 KO mouse brain, the infarct size, levels of pro-inflammatory markers and blood–brain barrier breakdown were found to be reduced, and the functional outcome was improved after focal cerebral ischemia compared to wild-type animals [36,38,39]. This would suggest that a pharmacological reduction of biologically active CCL2 has therapeutic potential in ischemic conditions and presumably also in other brain disorders with a neuroinflammatory component. This assumption is challenged by other studies demonstrating that deletion of the CCL2 receptor CCR2 contributes to an increased cerebral injury after ischemia [40,41,42]. However, in CCR2 KO tissue, CCL2 might still bind to CCR4 and mediate its effects via this receptor [43,44]. Therefore, we here investigated the contributions of the CCL2-stabilizing enzymes QC and isoQC on the functional outcome as well as cellular and molecular responses to ischemia. 

Our most interesting observation was an increased QC immunoreactivity and decreased isoQC immunoreactivity in brain regions affected by ischemia in wild-type mice. The increased expression of a protein by neurons affected by ischemia is surprising and may point to a very important cellular response to the ischemic insult. Like QC, the QC/isoQC substrate CCL2 was also induced in a time-dependent manner after ischemia in the wild-type mouse brain. In both the QC KO and isoQC KO mouse brain, the amount of CCL2 immunoreactivity was reduced by half. We detected CCL2 immunoreactivity frequently within glial cells, but also within neurons. Abolished neuronal QC/isoQC expression in KO mouse brains reduces CCL2 stabilization and thus its secretion into the extracellular space. Subsequently, glial CCL2 uptake via CCR2 or CCR4 is diminished as well.

Analyzing CCL2 protein by multiplex analysis showed a trend of reduced CCL2 in isoQC KO mice only. This indicated that isoQC is a major factor for stabilizing CCL2 as suggested by previous studies [20]. However, it also suggested that QC might be also a contributing factor for CCL2 stabilization. This point should be addressed in future studies in QC/isoQC double KO mice. CCL2 was found to be co-localized with a small proportion of Iba1-positive, MHC-II-positive and GFAP-positive glia cells and with neurons in the infarct area. However, from these immunohistochemical labeling it is not possible to discriminate between CCL2 expressed by or taken up into these cells. The appearance of CCL2 immunosignals as diffuse and spot-like extracellular labeling, and its association with blood vessels and glia cells, is in good agreement with CCL2 building up a chemotactic gradient starting by the adherence of CCL2 to the inner vessel wall as first point of contact with immature phagocytes.

A surprising observation was the time- and genotype-dependent effect of isoQC KO on CCL17, which was reduced in isoQC KO brain tissue, but not in QC KO brain tissue, compared to wild-type animals. CCL17 was originally described as murine thymus and activation-regulated chemokine (mTARC) that possesses chemotactic activity towards CD4^+^ T-cells [45]. CCL17 is not a QC/isoQC substrate since signal peptidase cleavage liberates an N-terminal alanine residue (Ala24) [45]. Therefore, it lacks an N-terminal glutamine residue, which is a prerequisite for a QC/isoQC-catalyzed N-terminal blockage by a pyroglutamate (pGlu) residue. Consequently, the observed reduction of CCL17 protein could be in relation to observed trends of reduced CX3CL1 and IL-1β in isoQC KO mice. In this case, immature CCL2 might prevent the full activation of the pro-inflammatory cytokine cascade following the ischemic insult. Since reports on CCL17 are scarce in the present literature, it is tempting to speculate that signal peptidase could also release mature CCL17 starting with glutamine at position 19 (Gln19). In this case, CCL17 could be an isoQC substrate and the observed reduction could be a direct effect of isoQC depletion. In particular, the presence of brain-specific CCL17 sharing the N-terminus with CX3CL1 as a result of alternative splicing [46] could point to a more complex biology of CCL17 than anticipated.

The cellular responses to ischemia include an instant activation of astrocytes and a delayed activation of Iba1-positive microglial cells and infiltration of MHC-II-positive cells, which coincides with increased QC and CCL2 expression levels. This is consistent with the activation and recruitment of Iba1- and MHC-II-positive cells via the QC/isoQC-CCL2 axis. We observed an up-regulation of the CD68-positive microglia sub-population and a down-regulation of the TMEM119-positive microglia sub-population after ischemia which is in line with numerous other studies investigating microglial cell activation after brain lesions [47,48].

Although we observed a regulation of QC, isoQC and their substrate CCL2 after ischemia and altered CCL2 protein levels in the QC KO and isoQC KO mouse brain, there were no strong differences regarding the survival rates, infarct volumes, and glial activation between the wild-type mice and the KO genotypes analyzed. There may be several explanations for this. First, the functions of QC and isoQC might be partly redundant so that the absence of one enzyme is compensated by the presence of the other. Second, both QCs have numerous substrates that might be affected by the lack of one enzyme or the other. Therefore, the functional outcome of experimental stroke is probably based on the pGlu stabilization of numerous proteins with a different impact in this process. Finally, although efforts have been made to consider a relatively long observation period in the study, the chosen period of 72 hours might have been too short to clearly delineate inflammatory consequences of ischemia, which are assumed to occur in the post-acute stage of ischemia [49].

Such a regulated expression of QC, but not isoQC, was already reported for thyroid carcinomas [50] and for Human Umbilical Vein Endothelial Cells (HUVECs) after stimulation with TNF-α/IL-1β [21]. However, it appears that this effect is cell type-specific since in human coronary artery smooth muscle cells (HCASMCs), a co-regulation of QC and isoQC after stimulation using TNF-α/IL-1β was observed [21].

## 5. Conclusions

We conclude that neuronal QC expression is induced in neurons of ischemic brain regions, leading to increased levels of its substrate CCL2. CCL2, in turn, was identified in subsets of neurons and glial cells and thus might contribute to microglia activation and MHC-II cell infiltration. This does not, however, affect the functional outcome of stroke in the experimental setting during the post-ischemic observation period of 72 h. Therefore, future studies should focus on long-term consequences of modulated CCL2 expression in ischemic stroke.

## Figures and Tables

**Table 1:** Primers used for genotyping.

Strain	Primer		Sequence 5‘ → 3‘	Product [bp]
QC KO	Pbd2-1	Common	GTC CGG TAA GGT GAG GAG AA	
	Pbd2-2	KO	TGA TGT GTG CGT TTC AGA GA	525 (KO)
	Pbd2-WT1	WT	CCA GAG ACA TCC TGG TAA AAC A	733 (WT)
		
isoQC KO	7b	Common	TGT CCA CCC TCC TAC CTC TC	212 + 106 (KO) (AvrII Digestion)
	8b	Common	TCA CCT GCT GCT TGA TTC TG	318 (WT) (AvrII Digestion)
		
CCL2 KO	oIMR7415	KO	GCC AGA GGC CAC TTG TGT AG	179 (KO)
	oIMR9219_opt	WT	TGA CAG TCC CCA GAG TCA CAT AG	287 (WT)
	oIMR9220_opt	Common	TCA TTG GGA TCA TCT TGC TGG TGA	

**Table 2:** Cocktails of primary antibodies used for double and triple labeling immunohistochemistry.

Primary Antibody		Dilution	Host	Company	Cat. No.	Detection
**single labeling**						
MHC-II		1:500	rat	Invitrogen	M5/114.15.2	Ni-DAB
NeuN		1:1000	mouse	Millipore	MAB 377	DAB
HuC/D		1:1000	mouse	Invitrogen	A-21271	DAB
QC		1:500	goat	IZI	77101	DAB/Ni-DAB
isoQC		1:200	rabbit	IZI	3285	DAB
**double labeling**						
NeuN		1:1000	mouse	Millipore	MAB 377	donkey anti-mouse Cy5
HuC/D +		1:1000	mouse	Invitrogen	A-21271	donkey anti-mouse Cy5
NFL		1:200	rabbit	Synaptic Systems	171002	donkey anti-rabbit Cy2
CCL2	+	1:200	goat	AF479	R&D	donkey anti-goat Cy3
	NeuN	1:1000	mouse	Millipore	MAB 377	donkey anti-mouse Cy5
	HuC/D	1:1000	mouse	Invitrogen	A-21271	donkey anti-mouse Cy5
	Hoechst	1:10,000	-	Invitrogen	H3570	-
	Iba1	1:500	rabbit	Wako	019-19741	donkey anti-rabbit Cy2
	CD68	1:100	rat	Biorad	MCA1957	donkey anti-rat Cy2
	TMEM119	1:100	rabbit	abcam	ab209064	donkey anti-rabbit Cy2
	GFAP	1:200	guinea pig	Synaptic Systems	173001	donkey anti-guinea pig Cy5
	Olig2	1:100	rabbit	abcam	ab81093	donkey anti-rabbit Cy5
**triple labeling**						
NeuN		1:1000	mouse	Millipore	MAB 377	donkey anti-mouse Cy3
HuC/D +		1:1000	mouse	Invitrogen	A-21271	donkey anti-mouse Cy3
Iba1 +		1:500	rabbit	Wako	019-19741	donkey anti-rabbit Cy2
GFAP		1:200	guinea pig	Synaptic Systems	173001	donkey anti-guinea pig Cy5

Secondary antibodies were all from Dianova and used at a dilution of 1:200.

## Data Availability

Original data, electronic image files and immunohistochemically labelled mouse brain tissue sections will be made available upon reasonable request.

## Supplementary Materials

The following supporting information can be downloaded at: https://www.mdpi.com/article/10.3390/cells13171412/s1, Figure S1: Specificity of antibodies against QC, isoQC and CCL2.
