# Supplementary material for: Focal Cerebral Ischemia Induces Expression of Glutaminyl Cyclase along with Downstream Molecular and Cellular Inflammatory Responses
Source: Cells. 2024 Aug 23;13(17):1412. doi: 10.3390/cells13171412 (PMC11394561; doi:10.3390/cells13171412)
Supplement: Supplementary file 1 [file cells-13-01412-s001.zip › cells-3139907-supplementary.pdf]

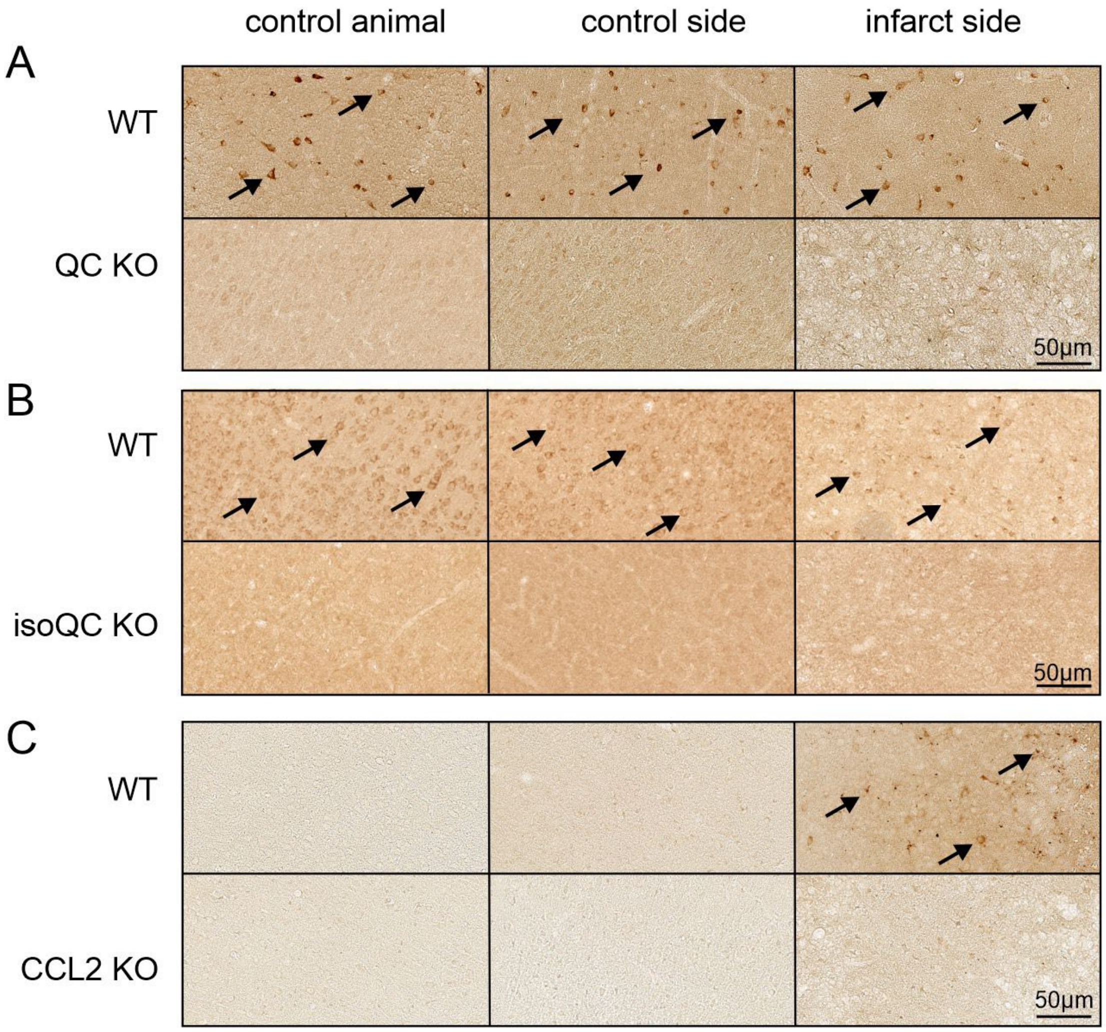

Supplementary Figure S1. Demonstration of the specificity of antibodies directed against glutaminyl cyclase (QC; A), isoglutaminyl cyclase (isoQC; B) and CCL2 (C) in cortex of wild type mice (WT) without ischemia (control animal) and in the respective knock-out mice (KO) in the control side and infarct side of mice with experimental stroke at 72 hours after ischemia. For details of immunohistochemical labellings see Methods section in the main text. **(A)** Note the labelling of individual QC-immunoreactive neurons (arrows) in all experimental conditions of the WT mouse (top) and the absence of QC immunoreactivity in the QC KO mouse (bottom). **(B)** Note the labelling of numerous isoQC-immunoreactive neurons (arrows) in all experimental conditions and reduced isoQC immunoreactivity in the infarct area of the WT mouse (top) and the absence of isoQC immunoreactivity in the isoQC KO mouse (bottom). **(C)** Note the absence of CCL2 labelling in the WT control animal without stroke and in the control side of a WT ischemic animal, but robust CCL2 immunoreactivity (arrows) in the infarct side of the WT animal (top). In the ischemic CCL2 KO mouse, no CCL2 immunoreactivity was detected (bottom).
